# EUPopLink Country report - Spain

**DOI:** 10.12688/openreseurope.23477.1

**Published:** 2026-04-16

**Authors:** Juan Roch, Luis Ramiro

**Affiliations:** 1 Universidad Autonoma de Madrid Departamento de Ciencia Politica y Relaciones Internacionales, Madrid, Community of Madrid, Spain; 2 Universidad Nacional de Educacion a Distancia- (UNED), Departamento de Ciencia Política y de la Administración, Community of Madrid, Spain

**Keywords:** Populism, Euroscepticism, Spain

## Abstract

This report examines the evolution of Spain’s relationship with Europe and the European Union (EU), as well as the rise and impact of populism and Euroscepticism within its political system. Following the democratic transition, European integration became a central pillar of Spain’s modernization and political legitimization, supported by a broad consensus among both elites and public opinion. Since joining the European Economic Community in 1986, support for the EU has remained consistently high, albeit sometimes characterized by limited public engagement. This consensus began to weaken after the 2008 financial crisis, which created opportunities for the emergence of new populist actors. In this context, Podemos on the radical left and Vox on the radical right gained prominence. Both parties incorporate elements of Euroscepticism, though in distinct ways. Podemos advances a form of soft Euroscepticism, criticizing neoliberal EU policies and political elites while maintaining a fundamentally positive view of the European project and advocating institutional reform. In contrast, Vox promotes a more nationalist and confrontational discourse, portraying EU elites as part of a “globalist” agenda and advocating a restructuring of the EU toward greater national sovereignty. Despite the rise of these challengers, mainstream parties—namely the PSOE and the PP—have maintained strong pro-European positions. Although they have adopted partially accommodative strategies in response to political competition, they have not significantly altered their support for European integration. Overall, Spain continues to exhibit a predominantly pro-EU political system and public opinion, where Euroscepticism, while increasingly visible, remains largely moderate in nature.

## 1. Introduction

The present country report was prepared as part of the EUPopLink COST Action ( Andreadis & Vasilopoulou, 2026) following the EUPopLink Theoretical Framework and guidelines ( Lorimer et al., 2026).

The relationship between Spain and the European “ideal” during and after the Francisco Franco dictatorship (1939–1975), and subsequently with the European Economic Community (EEC) and the European Union (EU), is crucial to understanding Spain’s political evolution following the democratic transition of the late 1970s. This trajectory, culminating in Spain’s accession to the EEC, shifted the country from relative isolation in Europe to a path of modernization and eventual full integration into the EU ( Boix, 2000: 166; Farrell, 2005: 151). Europe has served as a positive reference point since the early years of Spanish liberal democracy (from 1979 onward), although its idealization was already evident among segments of the intellectual elite before the end of the dictatorship. As Moreno (2013: 219) observes, Europe became a “master symbol” for most political actors during the early democratic period. This favourable perception intensified between the 1977 application for membership and Spain’s formal accession to the EEC in 1986.

A broad consensus existed within the Spanish party system regarding the benefits of joining the European Common Market, including the Communist Party, which in 1986 became part of the left-wing coalition Izquierda Unida (IU). According to Boix (2000: 166), party positions vis-à-vis the European Communities (EC) “merely reflected the overwhelming public support for the process of European integration” (see also Magone, 2016: 89; Avilés, 2004: 410). Between 1986 and 1989, positive evaluations of Spain’s EC membership rose from 62 percent to over 80 percent ( European Commission, 1986: 58; 1989: 9). However, Ruiz Jiménez and Egea de Haro (2011: 134) argue that this support rested on a “faulty Europeanism” as limited knowledge, indifference, and apathy remained persistent features of Spanish public opinion toward the EC/EU.

At the party-system level, the two main governing parties since 1982, the Social Democratic Partido Socialista Obrero Español (PSOE) and the conservative Partido Popular (PP), have consistently endorsed European integration, particularly following accession in 1986. EC membership fulfilled a long-standing objective of the political class and symbolized hope and democratic normalization for a significant portion of the population. Aside from fringe actors on the extreme left and right, the most notable Euroscepticism among nationwide parties emerged within IU, especially during the debates surrounding the Maastricht Treaty, though this position is generally classified as soft Euroscepticism ( Gómez-Reino et al., 2008).

This broad pro-EU consensus weakened in the aftermath of the 2008–2010 financial crisis and the subsequent political crises, which created fertile ground for the rise of new populist Eurosceptic actors in Spain. Nonetheless, both the party system and public opinion continue to display substantial support for the EU: nearly three-quarters of Spaniards (73.2%) consider membership “rather positive,” and 65.3% report being “very or quite satisfied” with forty years of Spain’s EU membership ( CIS, 2025).

## 2. Populist political actors

Two parties employing populist rhetoric deserve attention: Podemos on the radical left and Vox on the radical right. In the aftermath of the financial crisis, Podemos emerged as a new political actor, rising amid the decline of anti-austerity mobilizations that followed the 2011 15-M (Indignados) movement. For the first time, a populist party entered the Spanish political arena. During its early years, particularly before 2016, Podemos adopted a distinctly populist discourse, framing politics as a confrontation between “the people” and the elites, both political and economic, and being highly critical of the EU’s neoliberal policies ( Roch, 2022). At the national level, Podemos consolidated its position in the two general elections held in Spain within six months, first in December 2015 and then in June 2016 ( Rodríguez-Teruel et al., 2016).

While Podemos primarily grew on the left as a populist response to the Great Recession, the radical right Vox expanded predominantly on the right since 2018, as a nationalist and right-wing populist reaction to the secessionist movement in Catalonia. Vox was founded in 2013 but did not gain political representation until 2019. Despite some controversies about Vox’s populist nature, most scholars agree that the party exhibits certain populist traits, such as framing politics as a conflict between “the people” and “the elites”. However, this antagonism is not central to its discourse and nationalism and anti-immigration rhetoric tend to be more dominant ( Marcos-Marne et al., 2021; Ramos-González and Ortiz, 2022; Roch and Cordero, 2024)

Podemos’ and Vox’s political and electoral trajectories have been marked by considerable variation. In the most recent July 2023 general elections, Vox re-ran as a populist alternative to the PP, obtaining 33 seats, while Podemos had only five MPs in the overall 31 of the left-wing Sumar (Add Up) coalition. Sumar is a left-wing coalition formed for the July 2023 national election in response to the decline of previous radical left alliances led by Podemos. For the 2024 European elections, Sumar and Podemos ran separately, obtaining only three and two MPs, respectively. Together with the results achieved by Vox, these outcomes seem to indicate the consolidation of a multi-party system in which populist parties, both on the left and the right, play a fundamental role in shaping and facilitating government formation.

Podemos and Vox have both consistently shown signs of contestation of the main EU policies and institutional arrangements, although with significant differences and variations over time. Podemos is a radical-left party, whereas Vox is a radical-right party. Sumar appears to adopt a less Eurosceptic or Eurocritic stance than Podemos or at least tends to avoid politicizing EU-related issues or taking explicit positions on contentious European topics (see Haapala and Roch, 2025). Hence, this country report will focus on the degree and form of Euroscepticism of Podemos and Vox. The former is integrated in the European Parliament Group *The Left* and the latter is active part of the group Patriots for Europe, in which there are other prominent populist radical right parties such as Fidesz of Hungary or the Freedom Party of Austria.

## 3. Positions on Europe and the Populism-Euroscepticism nexus

### 3.1. The aftermath of the financial crisis and the rise of Podemos

Since the consolidation of Podemos in 2015, there was for the first time in Spain a prominent political party confronting the EU, its institutions and policies. Although the traditional radical left party in Spain (IU) was partially critical of EU neoliberal policies, it did not reach the electoral relevance of Podemos, and the diffusion of its positions was more limited. According to previous analyses of the initial stage of Podemos, the party exhibited confrontational rhetoric towards the EU, especially towards some of its leaders and institutions, such as Angela Merkel and the European Central Bank, which were seen as the main exponents of austerity politics in Europe ( Roch, 2021). However, Europe is represented positively, in contrast to the representation of EU institutions, policies, and political elites: “The problem is not Europe, the problem is that the president of the European Central Bank is called Mario Draghi and he was representative of Goldman Sachs in Europe […] Europe’s problem is called Durão Barroso […]” ( Podemos, 2014). ^1^ Podemos focuses on Germany, the European Central Bank and those responsible for austerity policies and portrayed them as *buriers* of the Europe of social rights that *should be recovered.* The problem of Europe is depicted as “a problem of its elites”, and Podemos proposes to bring back Europe to the people and to the southern European countries because “in a democracy the power has to be in the hands of the people” ( Podemos, 2015). Podemos argues Europe has been hijacked by political and economic elites. In this case, the populist discourse clearly shapes the representation of Europe by including it within the antagonistic divide between the legitimate people and the elites, positioning Europe as part of the former, on the positive side of “the people”.

The EU refugee crisis in 2015 had a limited impact on Spain, in part due to the political context marked by the rise of Podemos as a radical alternative to the bipartisan rotation of power between the centre-left PSOE and the centre-right PP. The lack of radical right parties with parliamentary seats was decisive in avoiding the politicization of migration issues and refugee policy, and a potential new avenue for the critique of the EU during the years 2015–16.

In 2016, Podemos merged with the radical left party IU in preparation for the general election in June 2016. In its role as an opposition party to the conservative People’s Party government, Podemos (and the coalition Unidas Podemos) toned down its negative references to the EU. The coalition increasingly adopted what can be called a “reformist approach” to the EU ( Roch, 2024), a version of soft Euroscepticism, characterized not by a wholesale rejection of the European project, but by targeted criticism of specific neoliberal EU policies. The EU is framed as a space where institutional reform is possible, even if its institutions are at times portrayed as being captured or undermined by elites. Podemos aim to build a Europe of more social rights and democracy: “Radically democratizing the EU institutions and the entire functioning of the EU in accordance with the following principles is the best way to prevent the EU from acting again against its peoples” ( Podemos, 2019). That Podemos and its ally IU were willing to combine their critical rhetoric with a pragmatic and full acceptance of the EU became evident over time. This moderate stance toward the EU became even more pronounced once the coalition government between Podemos (and IU) and the PSOE was born in January 2020.

After the general election of July 2023, Podemos was excluded from the five ministerial posts allocated to the left-wing coalition Sumar under the PSOE-Sumar coalition agreement. Soon after Podemos left the coalition government, broke with Sumar, and ran independently in the 2024 European elections. Since then, Podemos has pursued a strategy of radical left-wing differentiation. Podemos has attempted to distinguish itself from Sumar by adopting a more hardline stance on European Union and international policy issues. One of the key issues on which the Spanish radical left, Podemos and IU, has distanced itself from the EU policy consensus is the support for Ukraine in response to the Russian invasion. Both parties, especially IU, have expressed strong opposition to aiding Ukraine and have positioned themselves among the most radical voices within The Left group in the European Parliament on this matter ( Holesch et al. 2024). However, once Podemos left the Spanish government, they also used this issue to criticize not only the EU but also the PSOE–Sumar coalition government in Spain.

“Spain’s and the European Union’s stance on the war in Ukraine has only served to reinforce the interests of the United States, its global hegemony, and its business dealings, as it has become the world’s leading exporter of liquefied natural gas” ( Podemos, 2024)

### 3.2. The Euroscepticism of Vox since 2019

Vox’s primary focus has not been the European Union, but rather opposing the secessionist movement in Catalonia and promoting Spanish nationalism centred on the ideal of a strong nation and the figure of the “brave” and “authentic” Spaniard. However, in its broader crusade against left-wing politics and leftist elites, the EU was also portrayed as part of the problem. For instance, in Vox’s self-perception, the party presents itself as representing the middle and lower classes in opposition to the political “globalist” elites, as the following excerpt illustrates: “The so-called ‘green transitions’ consist of transferring huge amounts of money from the middle and working classes to the elites driving the climate agenda” ( Vox 2021). Again, rejecting what they call “the climate consensus”, Vox considers that “ecological fanaticism […] makes people poorer because they were the poorest and the weakest and those who needed most support” ( Vox, 2022).

Connected with these claims, the position of Vox on the EU has been radicalising in the last years, coming up to the 2024 European parliamentary elections. The EU elites were depicted as part of a coalition of interests contrary to Spaniards and the Spanish nation. In this vein, EU elites and the European Parliament passed legislation to restrain freedom and sovereignty of the European nations: “They have passed a European media law which means destroying media freedom and press freedom in Europe, they have passed a digital media law, a digital services law” ( Vox, 2024a). In Spain, the “old parties” and “systemic parties” ( Vox, 2024b), were portrayed as the actors supporting EU policies that endanger freedom and civil rights. Vox suggests clearly the connections between the EU, national mainstream parties, and the media. The problem is not only the “ *progre* [leftist] dictatorship” at the national level, but rather a coalition of interests tied to the EU, its policies, and the large-scale media serving those interests: “These media have been disseminating and promoting the 2030 Agenda for years. The 2030 Agenda continues to be disseminated and promoted by the IBEX companies, Big Banking, because apparently, they seem to be very nice words” ( Vox, 2024c). Vox portrays political elites in Brussels as those controlling the media and seeking to manipulate and ignore the “real” problems and concerns of the Spanish people:

“The mainstream media do not talk about the European elections. What do they want? What they want is for you to stay at home on June 9 and for them to continue with their grand coalition in Brussels, doing whatever they want, voting against the Spanish people and without any of the Spanish people finding out about it.”

( Vox, 2024c)

Vox advances a predominantly soft Eurosceptic position, seeking to reshape the European Union from within by transforming it into a looser coalition of fully sovereign nation-states with complete cultural and legal autonomy. In the 2024 European elections, a new radical right anti-establishment formation emerged in Spain: The Party is Over (Se Acabó La Fiesta, SALF), garnering three seats. This increases competition within the radical right over Euroscepticism and potentially triggers a struggle to claim ownership of opposition to the EU. The discourse of SALF is not explored in this country report due to the absence of a defined political program and the novelty and provisional nature of this emerging political formation.

## 4. Responses to Euroscepticism and consequences

Both the social democratic PSOE and the conservative PP faced strategic dilemmas and challenges stemming from the growing popularity of these more radical parties. Podemos and Vox expanded the political space by shifting the salience of key issue dimensions and attempting to claim ownership over certain policy areas at both the national and EU levels. The PSOE’s response to the challenge posed by Podemos in its own left-to-centre space included elements of accommodation, culminating in a coalition government that included ministers from both Podemos and IU. However, despite Podemos intensifying its criticism of EU policies, particularly in the aftermath of the austerity policies following the 2008 Euro crisis, the PSOE maintained a core element of its identity: its longstanding pro-European stance.

Despite increased competition from a left-wing Eurosceptic party, the PSOE has upheld a pro-EU platform advocating for deeper European integration. In socioeconomic terms, the PSOE has remained a moderate social democratic party, defending the European social model, economic regulation, redistributive policies, and the expansion of both the social pillar and civic rights. This pro-EU adversarial stance includes support for further integration and federalization, albeit sometimes vaguely expressed, through strengthening the roles of the European Parliament and the European Central Bank. Notably, the PSOE continued to defend this broadly pro-EU platform even when some incipient signs of public discontent with the EU became more visible during the Euro crisis ( Plaza-Colodro and Ramiro, 2021).

Similarly to the PSOE, the conservative PP is also a pro-EU party, expressing levels of support for European integration comparable to those of the Spanish social democrats. The similarity between the two parties extends to their responses to the rise of more vocally Eurosceptic radical-right and radical-left competitors. The PP’s reaction to the emergence of rivals in the right-to-centre political space—first the centre-liberal and notably Spanish nationalist Ciudadanos party, followed by the radical-right Vox—can be characterized as an accommodative yet polarizing strategy. PP leaders sought to compete directly with these two right-wing parties by adopting some of the positions of its competitors ( Rodríguez-Teruel, 2020). Traditionally, the PP had owned issues related to defending cultural traditionalism and conservative identity, including Spain’s traditional national identity. However, both Ciudadanos (temporarily) and Vox challenged the PP in several of these policy areas. While Ciudadanos targeted moderate right-wing voters with a territorial stance like that of the PP, Vox emerged as a distinctly more radical right-wing competitor.

However, this combination of accommodation and polarization met limits in the realm of EU politics and policy. As previously noted, the PP is fundamentally pro-EU. Aligned with the ideological positions of its European party family, the PP strongly supports European integration. Its conservative stances on contentious EU policy issues, such as immigration, defence, welfare, and environmental policy, do not undermine its overall pro-EU orientation. In this respect, neither the PSOE nor the PP have significantly or permanently altered their basic policy towards European integration in response to the entry of two radical, populist competitors on the right and left.

## 5. Short conclusion

Spain has one of the most pro-EU public opinions among the EU member states as well as a pro-EU party system, at least until 2015. Eurosceptic parties have had only a marginal presence, and when criticism of the EU did emerge, it was typically expressed in the form of soft Euroscepticism, as was the case with the radical left party IU. The two major mainstream parties, the PSOE and the PP, have both maintained pro-EU positions, reflecting the typical ideological nuances of center-left and conservative parties, respectively. However, this political landscape began to shift with the emergence of two radical-left and right parties—Podemos, founded in 2014, and Vox, founded in 2013 but gaining real political relevance only from 2019 onward.

While Podemos has adopted a clearly populist discourse, Vox has employed populist themes more ambiguously, though its rhetoric frequently exhibits populist traits. Although Podemos has experienced a decline in both organizational force and electoral support, Vox has shown greater electoral strength. Nonetheless, both parties remain, for the moment, significant actors within the Spanish party system. Notably, both parties combine their populist rhetoric with a degree of Euroscepticism: stronger in the case of Vox, and more moderate in the case of Podemos. However, what is particularly striking is that the rise of these populist and Eurosceptic forces has not led the mainstream parties to alter their pro-EU stance. While the PSOE and the PP have adopted somewhat accommodative strategies in other policy areas, they have consistently upheld their strong pro-European orientation despite the growing presence of Eurosceptic competitors.

## Ethics and consent

Ethical approval and consent were not required.

## Data Availability

No data associated with this article.

## Footnotes

^1^
J. M. Durão Barroso served as President of the European Commission from 2004 to 2014.
