# EUPopLink Country report - Spain

**DOI:** 10.12688/openreseurope.23477.2

**Published:** 2026-07-28

**Authors:** Juan Roch, Luis Ramiro

**Affiliations:** 1 Universidad Autonoma de Madrid Departamento de Ciencia Politica y Relaciones Internacionales, Madrid, Community of Madrid, Spain; 2 Universidad Nacional de Educación a Distancia- (UNED), Departamento de Ciencia Política y de la Administración, Community of Madrid, Spain

**Keywords:** Populism, Euroscepticism, Spain

## Abstract

This report examines the evolution of Spain’s relationship with Europe and the European Union (EU), focusing on how populism and Euroscepticism have developed within a political system that remains predominantly pro-European. Following the democratic transition, European integration became a central pillar of Spain’s modernization and political legitimization, supported by a broad consensus among both elites and public opinion. While this consensus was challenged during periods of economic and political crisis, support for European integration has remained comparatively resilient. After the 2008 financial crisis, Podemos on the radical left and Vox on the radical right incorporated elements of Euroscepticism, though in distinct ways. Podemos advances a form of soft Euroscepticism, criticizing neoliberal EU policies and political elites while maintaining a fundamentally positive view of the European project and advocating institutional reform. In contrast, Vox promotes a more nationalist and confrontational discourse, portraying EU elites as part of a “globalist” agenda and advocating a restructuring of the EU toward greater national sovereignty. Despite the rise of these challengers, mainstream parties—namely the PSOE and the PP—have maintained strong pro-European positions. This report shows that Euroscepticism in Spain is largely context-dependent and expressed mainly through criticism of specific EU policies or elites rather than through a rejection of European integration itself. Overall, Spain continues to exhibit a predominantly pro-EU party system and public opinion. Although populist actors have increased the visibility of Eurosceptic narratives, these remain largely moderate, episodic, and unlikely to challenge the broad societal and party-political consensus in favor of European integration.

## 1. Introduction

The present country report was prepared as part of the EUPopLink COST Action ( Andreadis & Vasilopoulou, 2026) following the EUPopLink Theoretical Framework and guidelines ( Lorimer *et al*., 2026). In line with these guidelines, the report focuses specifically on expressions of Euroscepticism articulated by populist and other political actors, rather than on populism as a broader phenomenon. Consequently, the analysis prioritizes those dimensions of party politics that relate to Europe and the European Union (EU), while broader features of the populist discourse are discussed only insofar as they are relevant to understanding parties’ positions on Europe and the EU.

Spain’s relationship with the idea of “Europe” during and after the Francisco Franco dictatorship (1939–1975), and subsequently with the European Economic Community (EEC) and the EU, is crucial to understanding its political evolution since the democratic transition. This trajectory, culminating in Spain’s accession to the EEC, shifted the country from relative isolation in Europe to a path of modernization and eventual full integration into the EU ( Boix, 2000: 166; Farrell, 2005: 151). Europe has served as a positive reference point since the early years of Spanish liberal democracy (from 1979 onward), although its idealization was already evident among segments of the intellectual elite before the end of the dictatorship. As Moreno (2013: 219) observes, Europe became a “master symbol” for most political actors during the early democratic period. This favourable perception intensified between the 1977 application for membership and Spain’s formal accession to the EEC in 1986.

A broad consensus existed within the Spanish party system regarding the benefits of joining the European Common Market, including the Communist Party, which in 1986 became part of the left-wing coalition Izquierda Unida (IU). According to Boix (2000: 166), party positions vis-à-vis the European Communities (EC) “merely reflected the overwhelming public support for the process of European integration” (see also Magone, 2016: 89; Avilés, 2004: 410). Between 1986 and 1989, positive evaluations of Spain’s EC membership among the public rose from 62 percent to over 80 percent ( European Commission, 1986: 58; 1989: 9). However, Ruiz Jiménez and Egea de Haro (2011: 134) argue that this support rested on a “faulty Europeanism” as limited knowledge, indifference, and apathy remained persistent features of Spanish public opinion toward the EC/EU.

At the party-system level, the two main governing parties since 1982—the Social Democratic Partido Socialista Obrero Español (PSOE) and the conservative Partido Popular (PP)—have consistently supported European integration, particularly since Spain’s accession in 1986. EC membership fulfilled a long-standing objective of the political elites and symbolized hope and democratic normalization for a significant portion of the population. Aside from fringe actors on the extreme left and right, the most notable Euroscepticism among nationwide parties emerged within IU, especially during the debates surrounding the Maastricht Treaty, though this position is generally classified as soft Euroscepticism ( Gómez-Reino *et al*., 2008).

In comparative perspective, Spain shares important similarities with Portugal and has often been regarded as part of an “Iberian exceptionalism”, characterized by sustained public and party support for European integration and, until recently, the absence of electorally successful populist radical-right parties ( Heyne & Manucci, 2021). The emergence of Vox has challenged this perception, bringing Spain closer to broader European patterns, while its orientations towards the EU has nevertheless remained more favorable than in many other European countries.

This broad pro-EU consensus weakened in the aftermath of the 2008–2010 financial crisis and the subsequent political crises, which created fertile ground for the rise of new populist Eurosceptic actors in Spain. Nonetheless, both the party system and public opinion continue to display substantial support for the EU: nearly three-quarters of Spaniards (73.2%) consider membership “rather positive,” and 65.3% report being “very or quite satisfied” with forty years of Spain’s EU membership ( CIS, 2025).

## 2. Populist political actors

Two parties employing populist rhetoric deserve attention: Podemos on the radical left and Vox on the radical right. Both parties are widely considered populist and Eurosceptic, as reflected in their classification in the PopuList ( Rooduijn *et al*., 2023). This report focuses on nationwide parties due to space constraints inherent to the country report format; therefore, regional parties are not systematically addressed, although they are acknowledged as relevant actors in the broader literature on populism and Euroscepticism in Spain ( Gómez-Reino *et al*., 2008). In the aftermath of the financial crisis, Podemos emerged as a new political actor, rising amid the decline of anti-austerity mobilizations that followed the 2011 15-M (Indignados) movement. For the first time, a populist party entered the Spanish political arena. During its early years, particularly before 2016, Podemos adopted a distinctly populist discourse, framing politics as a confrontation between “the people” and the elites, both political and economic, and being highly critical of the EU’s neoliberal policies ( Roch, 2022). Like Syriza in Greece, Podemos has been characterized as a new type of left-wing party that combines socialist and populist elements. Its ideological profile has also been linked to the influence of twenty-first-century Latin American left-wing populism, particularly during its early organizational formation and the development of its political discourse ( Kioupkiolis & Katsambekis, 2018). At the national level, Podemos consolidated its position in the two general elections held in Spain within six months, first in December 2015 and then in June 2016 ( Rodríguez-Teruel *et al*., 2016). In the July 2023 general election, Podemos won only five of the 31 seats secured by the left-wing coalition Sumar (“Add Up”). Sumar was formed ahead of the 2023 election in response to the decline of earlier radical-left alliances led by Podemos. In the 2024 European Parliament election, Sumar and Podemos contested separately, winning three and two seats, respectively.

While Podemos primarily grew on the left as a populist response to the Great Recession, the radical right Vox expanded predominantly on the right since 2018, as a nationalist and right-wing populist reaction to the secessionist movement in Catalonia. Founded in 2013, Vox remained electorally marginal for several years and did not gain parliamentary representation until the 2019 general election. The party’s emergence marked a significant departure from Spain’s long-standing reputation as an exception to the rise of the populist radical right in Europe ( Manucci, 2024). Its electoral breakthrough was facilitated by the heightened territorial conflict surrounding Catalan independence, dissatisfaction with the established conservative right, and the increasing salience of issues such as national identity, immigration, and law and order ( Rama *et al.*, 2021). Despite some controversies about Vox’s populist nature, most scholars agree that the party exhibits certain populist traits, such as framing politics as a conflict between “the people” and “the elites”. However, this antagonism is not central to its discourse and nationalism and anti-immigration rhetoric tend to be more dominant ( Marcos-Marne *et al*., 2021; Ramos-González and Ortiz, 2022; Roch and Cordero, 2023). In the July 2023 general elections Vox obtained 33 seats in the national parliament. Together with the results achieved by Podemos and Sumar, these outcomes appear to indicate the consolidation of a multi-party system in which populist parties on both the left and the right play an increasingly important role in shaping government formation.

Podemos and Vox have both consistently shown signs of contestation of the main EU policies and institutional arrangements, although with significant differences and variations over time. Podemos is a radical-left party, whereas Vox is a radical-right party. Sumar appears to adopt a less Eurosceptic or Eurocritic stance than Podemos or at least tends to avoid politicizing EU-related issues or taking explicit positions on contentious European topics (see Haapala and Roch, 2025). Hence, this country report will focus on the degree and form of Euroscepticism of Podemos and Vox. The former is integrated in the European Parliament Group *The Left* and the latter is active part of the group Patriots for Europe, in which there are other prominent populist radical right parties such as Fidesz of Hungary or the Freedom Party of Austria.

## 3. Positions on Europe and the Populism-Euroscepticism nexus

In line with the conceptual framework developed within the EUPopLink COST Action, this report understands populism as a political discourse or set of ideas centred on an antagonistic distinction between “the people” and “the elites”, combined with an appeal to popular sovereignty against unresponsive or corrupt elite rule ( Lorimer *et al*., 2026). This conceptualization draws on both the ideational and discursive approaches to populism, treating the people–elite antagonism and the appeal to popular sovereignty as its core defining features, while remaining agnostic on whether populism should be understood primarily as a thin-centered ideology or as a discursive frame. Consistent with this perspective, populism is not inherently exclusionary and can be combined with different host ideologies, giving rise to distinct variants such as populist radical-right, left-wing populist, or valence populist actors ( Lorimer *et al*., 2026).

Regarding Euroscepticism, this report adopts Paul Taggart’s (1998, p. 366) broad definition of the concept as “the idea of contingent or qualified opposition, as well as incorporating outright and unqualified opposition to the process of European integration” ( Lorimer *et al*., 2026). This conceptualization recognizes Euroscepticism as a dynamic and context-dependent phenomenon rather than a fixed party characteristic, allowing parties to adopt more or less critical positions towards the EU over time. Accordingly, the report distinguishes between *soft Euroscepticism*, which refers to qualified opposition to specific EU policies or institutional arrangements while accepting the broader project of European integration, and *hard Euroscepticism*, which denotes principled opposition to the EU or to European integration itself. To capture variation across parties, the analysis also considers the object, intensity, and nature of Eurosceptic positions ( Lorimer *et al*., 2026).

### 3.1 The aftermath of the financial crisis and the rise of Podemos

Since the consolidation of Podemos in 2015, there was for the first time in Spain a prominent political party confronting the EU, its institutions and policies. Although Spain’s traditional radical left party, IU, expressed criticism of EU neoliberal policies and adopted, at most, a soft Eurosceptic stance, it never attained the electoral relevance of Podemos, limiting the impact of its positions. Except for some peripheral nationalist radical left parties in certain regions, other far-left organizations in Spain, despite their hard Eurosceptic positions, lacked political significance. According to previous analyses of the initial stage of Podemos, the party exhibited confrontational rhetoric towards the EU, especially towards some of its leaders and institutions, such as Angela Merkel and the European Central Bank, which were seen as the main exponents of austerity politics in Europe ( Roch, 2021). However, Europe is represented positively, in contrast to the representation of EU institutions, policies, and political elites: “The problem is not Europe, the problem is that the president of the European Central Bank is called Mario Draghi and he was representative of Goldman Sachs in Europe […] Europe’s problem is called Durão Barroso […]” ( Podemos, 2014). ^1^ Podemos focused on Germany, the European Central Bank and those responsible for austerity policies and portrayed them as *buriers* of the Europe of social rights that *should be recovered.* The problem of Europe is depicted as “a problem of its elites”, and Podemos proposed to bring back Europe to the people and to the southern European countries because “in a democracy the power has to be in the hands of the people” ( Podemos, 2015). Podemos argues Europe has been hijacked by political and economic elites. In this case, the populist discourse clearly shapes the representation of Europe by including it within the antagonistic divide between the legitimate people and the elites, positioning Europe as part of the former, on the positive side of “the people”.

The EU refugee crisis in 2015 had only a limited impact on Spain, in part because political attention was focused on the emergence of Podemos as a radical alternative to the longstanding alternation in power between the centre-left PSOE and the centre-right PP. The absence of radical right parties in parliament was a key factor in preventing the politicization of migration and refugee policy, thereby limiting the emergence of a new avenue for criticism of the EU during the 2015–16 refugee crisis.

In 2016, Podemos formed an electoral alliance with the radical left party IU ahead of the June 2016 general election. In its role as an opposition party to the conservative People’s Party government, Podemos (and the coalition Unidas Podemos) toned down its negative references to the EU. The coalition increasingly adopted what can be called a “reformist approach” to the EU ( Roch, 2024), a version of soft Euroscepticism, characterized not by a wholesale rejection of the European project, but by targeted criticism of specific neoliberal EU policies. The EU is framed as a space where institutional reform is possible, even if its institutions are at times portrayed as being captured or undermined by elites. Podemos aim to build a Europe of social rights and democracy: “Radically democratizing the EU institutions and the entire functioning of the EU in accordance with the following principles is the best way to prevent the EU from acting again against its peoples” ( Podemos, 2019). That Podemos and its ally IU were willing to combine their critical rhetoric with a pragmatic and full acceptance of the EU became evident over time. This moderate stance toward the EU became even more pronounced once the coalition government between Podemos (and IU) and the PSOE was born in January 2020. This relative moderation in the party’s position towards the EU was accompanied by a broader decline in the salience of its populist discourse. While references to “the people” remained important, the antagonistic opposition between “the people” and “the elites” became less central, giving way to a more reformist and policy-oriented political discourse ( Roch, 2022). The moderation of Podemos’s EU position is supported by data from the Chapel Hill Expert Survey, although with some qualifications. Between 2014 and 2024, Podemos moderated its criticism of the EU, moving from a score of 4.4 in 2014 (on a 1–7 scale) to 5.3 in 2019 and 4.7 in 2024. This trajectory closely resembles that of the established radical left party IU, although the magnitude of the changes remains limited. Overall, both Podemos and IU can be classified as soft Eurosceptic parties, as their criticism is directed primarily towards specific EU policies and institutional arrangements rather than European integration itself.

Following the July 2023 general election, Podemos was excluded from the five ministerial posts allocated to the left-wing coalition Sumar under the PSOE–Sumar coalition agreement. Shortly thereafter, Podemos left the coalition, broke with Sumar, and contested the 2024 European Parliament election independently. Since then, Podemos has pursued a strategy of radical left-wing differentiation. Podemos has attempted to distinguish itself from Sumar by adopting a more hardline stance on European Union and international policy issues. One of the key issues on which the Spanish radical left, Podemos and IU, has distanced itself from the EU policy consensus is the support for Ukraine in response to the Russian invasion. Both parties have expressed strong opposition to aiding Ukraine and have positioned themselves among the most radical voices within The Left group in the European Parliament on this matter ( Holesch *et al*. 2024). However, following its departure from the Spanish government, Podemos increasingly used the issue to criticize both the EU and the PSOE–Sumar coalition government. As the party argued:

“Spain’s and the European Union’s stance on the war in Ukraine has only served to reinforce the interests of the United States, its global hegemony, and its business dealings, as it has become the world’s leading exporter of liquefied natural gas” ( Podemos, 2024)

### 3.2 The Euroscepticism of Vox since 2019

Vox’s primary focus has not been the European Union, but rather opposing the secessionist movement in Catalonia and promoting Spanish nationalism centred on the ideal of a strong nation and the figure of the “brave” and “authentic” Spaniard. However, in its broader crusade against left-wing politics and leftist elites, the EU was also portrayed as part of the problem. For instance, in Vox’s self-perception, the party presents itself as representing the middle and lower classes in opposition to the political “globalist” elites, as the following excerpt illustrates: “The so-called ‘green transitions’ consist of transferring huge amounts of money from the middle and working classes to the elites driving the climate agenda” ( Vox 2021). Again, rejecting what they call “the climate consensus”, Vox considers that “ecological fanaticism […] makes people poorer because they were the poorest and the weakest and those who needed most support” ( Vox, 2022).

Connected with these claims, the position of Vox on the EU has been radicalising in recent years, leading up to the 2024 European parliamentary elections. EU elites were depicted as part of a coalition of interests acting against the Spanish people and the Spanish nation, reflecting the characteristic populist construction of politics as a conflict between a virtuous people and corrupt or self-serving elites. In this vein, Vox interpreted that EU elites and the European Parliament passed legislation to restrain the freedom and sovereignty of the European nations: “They have passed a European media law which means destroying media freedom and press freedom in Europe, they have passed a digital media law, a digital services law” ( Vox, 2024a). In Spain, the “old parties” and “systemic parties” ( Vox, 2024b), were portrayed as the actors supporting EU policies that endangered freedom and civil rights. Vox clearly the pernicious connections between the EU, national mainstream parties, and the media. The problem is not only the “ *progre* [leftist] dictatorship” at the national level, but rather a coalition of interests tied to the EU, its policies, and the large-scale media serving those interests: “These media have been disseminating and promoting the 2030 Agenda for years. The 2030 Agenda continues to be disseminated and promoted by the IBEX companies, Big Banking, because apparently, they seem to be very nice words” ( Vox, 2024c). Vox portrays political elites in Brussels as those controlling the media and seeking to manipulate and ignore the “real” problems and concerns of the Spanish people:

“The mainstream media do not talk about the European elections. What do they want? What they want is for you to stay at home on June 9 and for them to continue with their grand coalition in Brussels, doing whatever they want, voting against the Spanish people and without any of the Spanish people finding out about it.” ( Vox, 2024c)

Vox advances a predominantly soft Eurosceptic position, seeking to reshape the European Union from within by transforming it into a looser coalition of fully sovereign nation-states with complete cultural and legal autonomy. Vox is, in any case, the most Eurosceptic among Spain’s electorally relevant and politically significant parties, a position consistently confirmed by Chapel Hill Expert Survey data. In the CHES surveys, Vox receives scores of 3.2 in 2019 and 3.1 in 2024 on the 1–7 EU position scale, placing it clearly below other major Spanish parties in terms of support for European integration. In the 2024 European elections, a new radical right anti-establishment formation emerged in Spain: The Party is Over (Se Acabó La Fiesta, SALF), garnering three seats. This increases competition within the radical right over Euroscepticism and potentially triggers a struggle to claim ownership of opposition to the EU. The discourse of SALF is not explored in this country report due to the absence of a defined political program and the novelty and provisional nature of this emerging political formation.

## 4. Responses to Euroscepticism and consequences

Both the social democratic PSOE and the conservative PP faced strategic dilemmas and challenges stemming from the growing popularity of these more radical parties. Podemos and Vox expanded the political space by shifting the salience of key issue dimensions and attempting to claim ownership over certain policy areas at both the national and EU levels. The PSOE’s response to the challenge posed by Podemos in its own left-to-centre space included elements of accommodation, culminating in a coalition government that included ministers from both Podemos and IU. However, despite Podemos intensifying its criticism of EU policies, particularly in the aftermath of the austerity policies following the 2008 Euro crisis, the PSOE maintained a core element of its identity: its longstanding pro-European stance.

Despite increased competition from a left-wing Eurosceptic party, the PSOE has upheld a pro-EU platform advocating for deeper European integration. In socioeconomic terms, the PSOE has remained a moderate social democratic party, defending the European social model, economic regulation, redistributive policies, and the expansion of both the social pillar and civic rights. This pro-EU adversarial stance includes support for further integration and federalization, albeit sometimes vaguely expressed, through strengthening the roles of the European Parliament and the European Central Bank. Notably, the PSOE continued to defend this broadly pro-EU platform even when some incipient signs of public discontent with the EU became more visible during the Euro crisis ( Plaza-Colodro and Ramiro, 2021).

Similarly, to the PSOE, the conservative PP is also a pro-EU party, expressing levels of support for European integration comparable to those of the Spanish social democrats. The similarity between the two parties extends to their responses to the rise of more vocally Eurosceptic radical-right and radical-left competitors. The PP’s reaction to the emergence of rivals in the right-to-centre political space—first the centre-liberal and notably Spanish nationalist Ciudadanos party, followed by the radical-right Vox—can be characterized as an accommodative yet polarizing strategy. PP leaders sought to compete directly with these two right-wing parties by adopting some of the positions of its competitors ( Rodríguez-Teruel, 2020). Traditionally, the PP had owned issues related to defending cultural traditionalism and conservative identity, including Spain’s traditional national identity. However, both Ciudadanos (temporarily) and Vox challenged the PP in several of these policy areas. While Ciudadanos sought to attract moderate right-wing voters by adopting a territorial stance similar to that of the PP, Vox emerged as a distinctly more radical right-wing challenger.

However, this combination of accommodation and polarization met limits in the realm of EU politics and policy. As previously noted, the PP is fundamentally pro-EU. Aligned with the ideological positions of its European party family, the PP strongly supports European integration. Its conservative stances on contentious EU policy issues, such as immigration, defence, welfare, and environmental policy, do not undermine its overall pro-EU orientation. In this respect, neither the PSOE nor the PP have significantly or permanently altered their basic policy towards European integration in response to the entry of two radical, populist competitors on the right and left. This continuity may also reflect the incentives faced by parties with governing ambitions to preserve their credibility as responsible actors in both domestic and European politics. Given the persistently high levels of public support for European integration in Spain, abandoning a pro-European stance would also entail considerable electoral and reputational costs.

## 5. Short conclusion

Spain has one of the most pro-EU public opinions among the EU member states as well as a pro-EU party system, at least until 2015. Eurosceptic parties have had only a marginal presence, and when criticism of the EU did emerge, it was typically expressed in the form of soft Euroscepticism, as was the case with the radical left party IU. The two major mainstream parties, the PSOE and the PP, have both maintained pro-EU positions, reflecting the typical ideological nuances of center-left and conservative parties, respectively. However, this political landscape began to change with the emergence of two radical-left and right parties—Podemos, founded in 2014, and Vox, founded in 2013 but gaining real political relevance only from 2019 onward.

While Podemos has adopted a clearly populist discourse, Vox has employed populist themes more ambiguously, though its rhetoric frequently exhibits populist traits. Whereas Podemos has experienced a sustained decline in both organizational capacity and electoral support, becoming a much less influential political actor, Vox has consolidated its electoral position and remains one of the main challengers to the mainstream parties. Notably, both parties combine their populist rhetoric with a degree of Euroscepticism: stronger in the case of Vox, and more moderate in the case of Podemos. However, the rise of these populist and Eurosceptic forces has not led the mainstream parties to alter their pro-EU stance. While the PSOE and the PP have adopted somewhat accommodative strategies in other policy areas, they have consistently upheld their strong pro-European orientation despite the growing presence of Eurosceptic competitors.

## Ethics and consent

Ethical approval and consent were not required.

## Data Availability

No data associated with this article.

## Footnotes

^1^
J. M. Durão Barroso served as President of the European Commission from 2004 to 2014.
