# Supplementary material for: EUPopLink Country report - Spain
Source: Open Res Eur. 2026 Jul 28;6:114. Originally published 2026 Apr 16. [Version 2] doi: 10.12688/openreseurope.23477.2 (PMC13273243; doi:10.12688/openreseurope.23477.2)
Supplement: Peer review history

# Reviewer report: Reviewer response for version 2

**DOI:** 10.21956/openreseurope.26601.r78120

**Published:** 2026-07-29

**Authors:** Vít Hloušek

**Affiliations:** 1 Metropolitan University Prague, Praha, Czech Republic

I do not have any new comments.

Is the case presented with sufficient detail to be useful for teaching or other practitioners?

Partly

Is the work clearly and accurately presented and does it cite the current literature?

Yes

If applicable, is the statistical analysis and its interpretation appropriate?

Not applicable

Are all the source data underlying the results available to ensure full reproducibility?

No source data required

Are the conclusions drawn adequately supported by the results?

Yes

Is the background of the case’s history and progression described in sufficient detail?

Yes

Reviewer Expertise:

Euroscepticism, party politics, contemporary history, Central European politics

I confirm that I have read this submission and believe that I have an appropriate level of expertise to confirm that it is of an acceptable scientific standard.

# Reviewer report: Reviewer response for version 1

**Published:** 2026-06-16

**Authors:** Ana Belchior, Filipe Pacheco Silva

**Affiliations:** 1 ISCTE-IUL, Instituto Universitário de Lisboa, Lisboa, Portugal; 2 ISCTE-IUL, Instituto Universitário de Lisboa, Portugal, Portugal

This is a pleasant-to-read and historically informative country report focusing on Euroscepticism and populism in the Spanish party system. In our view, however, there is still room for improvement. We recommend that some revisions be undertaken and also offer a few additional minor suggestions for the authors’ consideration. Our comments are listed below.

 1. A core concern relates to the balance between the treatment of populism and Euroscepticism, given that the latter clearly dominates the report. Although Euroscepticism may have a stronger and longer presence in party discourse than populism, or there may simply be more information available on this topic, if the purpose of the report is to integrate both concepts, a greater effort should be made to ensure a more balanced treatment of each. Alternatively, the authors could explicitly acknowledge that Euroscepticism has been more relevant within the Spanish party system.

 2. Another concern relates to regional parties. Since these two phenomena are not confined to Spanish national parties (e.g., Gómez-Reino et al., 2008), the authors should either explicitly acknowledge that the report focuses exclusively on national parties and therefore excludes regional parties, or they should also address Euroscepticism and populism among regional parties.

 3. We agree with the other two reviewers regarding the need for greater conceptual clarification and a clearer presentation of the dimensions underlying both concepts. For instance, the classification of parties as soft Eurosceptic requires at least a brief explanation of the soft–hard Euroscepticism dichotomy. Furthermore, if the authors choose to characterize one party in these terms, they should also position the other parties within the same framework (and maybe clarify that, in most cases, what is at stake when Euroscepticism is discussed is dissatisfaction with the performance of EU institutions rather than support for Spain's withdrawal from the EU). The same applies to populism and to the dimensions commonly associated with it in the literature, particularly in the work of Cas Mudde.

 4. The discussion of parties’ positions could also benefit from incorporating evidence from the Chapel Hill Expert Survey (CHES) on European integration and populism. For example, CHES data suggest some evolution in Podemos’ stance towards European integration over time, more clearly indicating changes in the salience attached to this issue. European integration was most salient for Podemos in 2014, declining thereafter. This pattern suggests that the politicisation of European integration was, at least in part, a strategic response to the context of the financial crisis and the political opportunities it created for a radical left populist challenger. As Blyth and Hopkin (2018) argue, the Eurozone crisis particularly favoured the emergence of radical left populist parties in Southern Europe.

 In the same vein, CHES data show VOX emerging as the Spanish party with the least favourable position towards European integration in recent years. Although the authors mention this in the conclusion, introducing this evidence earlier in the analysis would help illustrate the extent to which each of the two parties (Podemos and VOX) question European integration. Doing so would also allow for a clearer comparison between the different forms of Euroscepticism expressed by these parties. The same applies to populism.

 5. The PopuList could also be used as a consensual data source for classifying parties as populist and Eurosceptic (Rooduijn et al., 2024).

 6. A final concern relates to how the Spanish party system is situated in comparison with other European countries, especially Southern European countries, and more particularly Portugal. For some time, scholars referred to an Iberian exceptionalism regarding the absence of parliamentary representation of the populist radical right in Spain and Portugal (see, among others, Heyne and Manucci, 2021). Likewise, Portugal and Spain followed a very similar pattern in terms of party support and fluctuations in public opinion towards the EU after accession. Situating Spain within a broader comparative framework would make it possible to assess the extent to which its trajectory conforms to, or diverges from, patterns observed in other European countries.

 Other less central points:

 - The abstract could be refined to better frame the main argument regarding the presence of populism and Euroscepticism in the Spanish political system, avoiding the introduction of too many details on the historical development of both phenomena. Otherwise, the abstract reads more as a summary of the entire report, repeating what is stated later in the text. For instance, the authors could draw on the argument that Euroscepticism in Spain is mainly ephemeral and context-dependent, as suggested by several authors (e.g., Parker & Tsarouhas, 2018; Teperoglou & Belchior, 2020; or on the pandemic crisis: Belchior et al., 2026), or develop another framing that better synthesises the report’s main argument.

 - When episodes that function as historical milestones are mentioned in the report, it would be clearer if the respective dates were also included alongside them.

 - We recommend removing non-fully intuitive expressions such as “European ‘ideal’”, or clarifying what is meant by them, in order to avoid any ambiguity.

 - There is some redundancy in the development of the argument that could be reduced.

 - Finally, regarding how both mainstream parties have responded to populist challenges, the authors show that, despite ideological nuances, both the PSOE and PP have adopted an accommodative approach. With regard to their stance on EU integration, the authors clearly demonstrate that both parties not only maintained their positions but even continued to advocate for the deepening of European integration. In highlighting this aspect, the authors appear to suggest that this outcome was somewhat expected, and that it did not materialise. As they note, “however, what is particularly striking is that the rise of these populist and Eurosceptic forces has not led the mainstream parties to alter their pro-EU stance”. It may also be worth considering the possibility that, by maintaining their commitment to EU integration, the PSOE and PP seek to preserve their image as responsible governing parties, without jeopardising their position among electorates that continue to support the European project.

**References:**

 Reference 1

 Reference 2

 Reference 3

 Reference 4

 Gómez-Reino M, Llamazares I and Ramiro L (2008) Euroscepticism and political parties in Spain. In: Szczerbiak A and Taggart P (eds) *Opposing Europe? The Comparative Party Politics of Euroscepticism volume 1: Case Studies and Country Surveys.* Oxford: Oxford University Press, pp.134–151.

 Parker, O., & Tsarouhas, D. (Eds.). (2018). *Crisis in the Eurozone periphery, The political economies of Greece, Spain, Ireland and Portugal*. Springer.

 Rooduijn, Matthijs, Andrea L.P. Pirro, Daphne Halikiopoulou, Caterina Froio, Stijn van Kessel, Sarah L. de Lange, Cas Mudde, and Paul Taggart (2024). The PopuList: A Database of Populist, Far-Left, and Far-Right Parties Using Expert-Informed Qualitative Comparative

 Classification (EiQCC). *British Journal of Political Science*, 1–10 **.**

Is the case presented with sufficient detail to be useful for teaching or other practitioners?

Partly

Is the work clearly and accurately presented and does it cite the current literature?

Yes

If applicable, is the statistical analysis and its interpretation appropriate?

Not applicable

Are all the source data underlying the results available to ensure full reproducibility?

No source data required

Are the conclusions drawn adequately supported by the results?

Partly

Is the background of the case’s history and progression described in sufficient detail?

Yes

Reviewer Expertise:

Political Science; including Euroscepticism and Populism

We confirm that we have read this submission and believe that we have an appropriate level of expertise to confirm that it is of an acceptable scientific standard, however we have significant reservations, as outlined above.

## References

[1] : Euroscepticism in party discourse during the COVID-19 pandemic: Evidence from Portugal and Spain. *European Union Politics* .2026;27(2) : 10.1177/14651165261424975 373-394 10.1177/14651165261424975

[2] : The Global Economics of European Populism: Growth Regimes and Party System Change in Europe (TheGovernment and Opposition /Leonard Schapiro Lecture 2017). *Government and Opposition* .2019;54(2) : 10.1017/gov.2018.43 193-225 10.1017/gov.2018.43

[3] : A new Iberian exceptionalism? Comparing the populist radical right electorate in Portugal and Spain. *Political Research Exchange* .2021;3(1) : 10.1080/2474736X.2021.1989985 10.1080/2474736X.2021.1989985

[4] : Ιs ‘Old Southern Europe’ Still Eurosceptic? Determinants of Mass Attitudes before, during and after the Eurozone Crisis. *South European Society and Politics* .2024;29(4) : 10.1080/13608746.2020.1805878 405-430 10.1080/13608746.2020.1805878

# Response

**Published:** 2026-07-09

**Authors:** Juan Roch

**Affiliations:** Political Science and International Relations, Autonomous University of Madrid Department of Political Science and International Relations, Madrid, Community of Madrid, Spain

Thank you for your comments. Below, we provide a point-by-point response to the issues raised.     This is a pleasant-to-read and historically informative country report focusing on Euroscepticism and populism in the Spanish party system. In our view, however, there is still room for improvement. We recommend that some revisions be undertaken and also offer a few additional minor suggestions for the authors’ consideration. Our comments are listed below.

 1. A core concern relates to **the balance between the treatment of populism and Euroscepticism, given that the latter clearly dominates the report.** Although Euroscepticism may have a stronger and longer presence in party discourse than populism, or there may simply be more information available on this topic, if the purpose of the report is to integrate both concepts, a greater effort should be made to ensure a more balanced treatment of each. Alternatively, the authors could explicitly acknowledge that Euroscepticism has been more relevant within the Spanish party system.

 Thank you for this thoughtful comment. We believe this point is largely related to the methodological guidelines provided for the preparation of the EUPopLink country reports, which are intended to ensure coherence across the collection. These guidelines explicitly state that the reports “should not be about populism and its different ideological forms as such, if there is no clear connection to the question of ‘Europe’”, with the main emphasis placed on populist (and other) expressions of Euroscepticism and political actors’ positions towards the EU. We have attached the relevant guidelines for reference, as we appreciate that reviewers were not aware of this document. In the revised manuscript we now clarify this analytical focus in the introduction and, nevertheless, where appropriate, we place greater emphasis on the populist dimension of Podemos and Vox in the empirical section.

 2. Another concern relates to regional parties. Since these two phenomena are not confined to Spanish national parties (e.g., Gómez-Reino et al., 2008), the authors **should either explicitly acknowledge that the report focuses exclusively on national parties and therefore excludes regional parties**, or they should also address Euroscepticism and populism among regional parties.

 We thank the reviewer for this valuable observation. We fully agree that regional parties in Spain are relevant actors in both populist and Eurosceptic dynamics. However, this report follows the format and length constraints of the Open Research Europe country report template (approximately 3,000 words excluding references), which requires a focused analytical scope. Given these limitations, the report concentrates on nationwide parties in order to allow a coherent and systematic discussion of the interaction between populism and Euroscepticism at the state-wide party-system level. This limitation has now been explicitly acknowledged in the revised manuscript.

 3. We agree with the other two reviewers regarding the need **for greater conceptual clarification and a clearer presentation of the dimensions underlying both concepts**. For instance, the classification of parties as soft Eurosceptic requires at least **a brief explanation of the soft–hard Euroscepticism dichotomy.** Furthermore, if the authors choose to characterize one party in these terms, they should also position the other parties within the same framework (and maybe clarify that, in most cases, what is at stake when Euroscepticism is discussed is dissatisfaction with the performance of EU institutions rather than support for Spain's withdrawal from the EU). The same applies to populism and to the dimensions commonly associated with it in the literature, particularly in the work of Cas Mudde.

 We thank the reviewer for this helpful suggestion. In response, we have introduced a conceptual section to clarify the definitions of populism and Euroscepticism adopted in the report, following the EUPopLink Theoretical Framework (Lorimer et al., 2026). In particular, we now briefly explain the distinction between soft and hard Euroscepticism and the conceptualisation of populism underpinning our analysis. We have also refined the classification and discussion of the main political parties to ensure that it is applied consistently in line with these conceptual definitions.

 4. The discussion of parties’ positions could also benefit from incorporating evidence from the Chapel Hill Expert Survey (CHES) on European integration and populism. For example, CHES data suggest some evolution in Podemos’ stance towards European integration over time, more clearly indicating changes in the salience attached to this issue. European integration was most salient for Podemos in 2014, declining thereafter. This pattern suggests that the politicisation of European integration was, at least in part, a strategic response to the context of the financial crisis and the political opportunities it created for a radical left populist challenger. As Blyth and Hopkin (2018) argue, the Eurozone crisis particularly favoured the emergence of radical left populist parties in Southern Europe.

 In the same vein, CHES data show VOX emerging as the Spanish party with the least favourable position towards European integration in recent years. Although the authors mention this in the conclusion, introducing this evidence earlier in the analysis would help illustrate the extent to which each of the two parties (Podemos and VOX) question European integration. Doing so would also allow for a clearer comparison between the different forms of Euroscepticism expressed by these parties. The same applies to populism.

 ​ Thank you very much for this suggestion. We have added the CHES scores regarding Podemos and Vox in sections 3.1 and 3.2.

 5. The PopuList could also be used as a consensual data source for classifying parties as populist and Eurosceptic (Rooduijn et al., 2024).

 Thank you very much for this suggestion. We have added a sentence and a reference to the PopuList at the beginning of Section 2.

 6. A final concern relates to how the Spanish party system is situated in comparison with other European countries, especially Southern European countries, and more particularly Portugal. For some time, scholars referred to an Iberian exceptionalism regarding the absence of parliamentary representation of the populist radical right in Spain and Portugal (see, among others, Heyne and Manucci, 2021). Likewise, Portugal and Spain followed a very similar pattern in terms of party support and fluctuations in public opinion towards the EU after accession. Situating Spain within a broader comparative framework would make it possible to assess the extent to which its trajectory conforms to, or diverges from, patterns observed in other European countries.

  We thank the reviewer for this valuable suggestion. In response, we have strengthened the introduction by situating the Spanish case within a broader comparative European perspective. Specifically, we now refer to the notion of “Iberian exceptionalism”, highlighting the similarities between Spain and Portugal in terms of their traditionally high levels of support for European integration and the historically limited electoral success of the populist radical right. We also clarify that the emergence of Vox has challenged this pattern, bringing Spain closer to broader European developments.  

 Other less central points:

 - The abstract could be refined to better frame the main argument regarding the presence of populism and Euroscepticism in the Spanish political system, avoiding the introduction of too many details on the historical development of both phenomena. Otherwise, the abstract reads more as a summary of the entire report, repeating what is stated later in the text. For instance, the authors could draw on the argument that Euroscepticism in Spain is mainly ephemeral and context-dependent, as suggested by several authors (e.g., Parker & Tsarouhas, 2018; Teperoglou & Belchior, 2020; or on the pandemic crisis: Belchior et al., 2026), or develop another framing that better synthesises the report’s main argument.

 We thank the reviewer for this helpful suggestion. In response, we have revised the abstract to place greater emphasis on the report’s main argument rather than on the historical development of populism and Euroscepticism. The revised version now highlights more explicitly that, despite the growing visibility of populist actors, Euroscepticism in Spain remains largely contextual, moderate, and linked to specific political and economic circumstances rather than constituting a sustained challenge to European integration. We have also streamlined some of the historical background in order to avoid repetition and improve the focus of the abstract.

 - When episodes that function as historical milestones are mentioned in the report, it would be clearer if the respective dates were also included alongside them.

 - We recommend removing non-fully intuitive expressions such as “European ‘ideal’”, or clarifying what is meant by them, in order to avoid any ambiguity.

 - There is some redundancy in the development of the argument that could be reduced.

 - Finally, regarding how both mainstream parties have responded to populist challenges, the authors show that, despite ideological nuances, both the PSOE and PP have adopted an accommodative approach. With regard to their stance on EU integration, the authors clearly demonstrate that both parties not only maintained their positions but even continued to advocate for the deepening of European integration. In highlighting this aspect, the authors appear to suggest that this outcome was somewhat expected, and that it did not materialise. As they note, “however, what is particularly striking is that the rise of these populist and Eurosceptic forces has not led the mainstream parties to alter their pro-EU stance”. It may also be worth considering the possibility that, by maintaining their commitment to EU integration, the PSOE and PP seek to preserve their image as responsible governing parties, without jeopardising their position among electorates that continue to support the European project.

   Thanks for these minor suggestions. We have changed the expression ideal at the beginning of the report. We thank the reviewer for these helpful suggestions. We have addressed all four points in the revised manuscript. We have removed or clarified potentially ambiguous expressions, such as “European ideal”. We have also streamlined several sections of the manuscript to reduce redundancy. Finally, we have expanded the discussion of the PSOE’s and PP’s responses to populist challengers by acknowledging that their continued commitment to European integration may also reflect their incentives to maintain credibility as responsible governing parties in a context of persistently high public support for the EU.

# Reviewer report: Reviewer response for version 1

**DOI:** 10.21956/openreseurope.25410.r74401

**Published:** 2026-05-22

**Authors:** Vít Hloušek

**Affiliations:** 1 Metropolitan University Prague, Praha, Czech Republic

The article describes and analyses the current Eurosceptic actors in Spain. The author focuses on Podemos and Vox, and sets the current Eurosceptic actors into a broader context. In my view, the paper is clearly written, logically structured and it allows even less informed readers to grasp the nature and versions of the current Spanish Euroscepticism.

 I suggest only two things to be briefly addressed in the revised version.

 First, although the author cites the general framework of the the EUPopLink COST Action project, where the concept of Euroscepticism is delimited, it would be more than useful to spend a brief paragraph demonstrating clearly what concept of Euroscepticism the author follows and what are the basic features of soft and hard Euroscepticism.

 Second, in the part devoted to Podemos, it would be useful to present briefly the stances of of the Spanish far left parties to the EU.

Is the case presented with sufficient detail to be useful for teaching or other practitioners?

Partly

Is the work clearly and accurately presented and does it cite the current literature?

Yes

If applicable, is the statistical analysis and its interpretation appropriate?

Not applicable

Are all the source data underlying the results available to ensure full reproducibility?

No source data required

Are the conclusions drawn adequately supported by the results?

Yes

Is the background of the case’s history and progression described in sufficient detail?

Yes

Reviewer Expertise:

Euroscepticism, party politics, contemporary history, Central European politics

I confirm that I have read this submission and believe that I have an appropriate level of expertise to confirm that it is of an acceptable scientific standard.

# Response

**Published:** 2026-07-09

**Authors:** Juan Roch

**Affiliations:** Political Science and International Relations, Autonomous University of Madrid Department of Political Science and International Relations, Madrid, Community of Madrid, Spain

Thank you for your comments. Below, we provide a point-by-point response to the issues raised.    

 The article describes and analyses the current Eurosceptic actors in Spain. The author focuses on Podemos and Vox, and sets the current Eurosceptic actors into a broader context. In my view, the paper is clearly written, logically structured and it allows even less informed readers to grasp the nature and versions of the current Spanish Euroscepticism.

 I suggest only two things to be briefly addressed in the revised version.

 First, although the author cites the general framework of the the EUPopLink COST Action project, where the concept of Euroscepticism is delimited, it would be more than useful to spend a brief paragraph demonstrating clearly what concept of Euroscepticism the author follows and what are the basic features of soft and hard Euroscepticism.

*We thank the reviewer for this helpful suggestion. In response, we have expanded the conceptual framework by briefly clarifying the definitions of both populism and Euroscepticism adopted in the report, following the EUPopLink COST Action framework (Lorimer et al., 2026). We now explicitly define Euroscepticism, distinguish between its soft and hard forms, and clarify the conceptual approach to populism used throughout the analysis*

 Second, in the part devoted to Podemos, it would be useful to present briefly the stances of of the Spanish far left parties to the EU.

*Thank you very much for this comment. We have briefly expanded on this point in a sentence added at the beginning of Section 3.1, “The aftermath of the financial crisis and the rise of Podemos.”*

# Reviewer report: Reviewer response for version 1

**DOI:** 10.21956/openreseurope.25410.r72537

**Published:** 2026-04-30

**Authors:** Ezgi Elçi

**Affiliations:** 1 Özyeğin University, Istanbul, Turkey

This country report examines the current populist actors in Spanish politics: Podemos and Vox. Please find my comments below, which, I hope, will be helpful to the authors.

 Theoretically, populism has three subdimensions: people-centrism, anti-elitism, and the Manichean outlook (Mudde, 2007). Euroscepticism can be located under anti-elitism, where European elites are depicted as self-interested outsider elites working against the pure (Spanish) people (Taggart, 1998). Both right- and left-wing populist parties carry these three characteristics. In addition, while populist radical right parties embrace populism, nativism, and authoritarianism (Mudde, 2019), populist radical left parties support a variant of socialism in which the focus on the proletariat is replaced by the vox populi (March & Mudde, 2005).

 Considering these theoretical approaches, the authors’ examination can be strengthened in several ways. First, Vox is depicted solely as a nationalist actor, which weakens its populist radical right characteristics (Rama et al., 2021). Second, the authors did not explain Vox’s rise as in-depth as Podemos’ historical trajectory. Third, the relevance of Podemos in coalition politics is explained on page 4, which can be explained earlier. Fourth, like Syriza, Podemos is a new type of party that blends socialism and populism, and is also related to discussions of 21st-century Latin American populism (Kioupkiolis & Katsambekis, 2017). Finally, once depicted as immune to exclusionary populism, Vox represents a deviation, like Chega in Portugal (Manucci, 2024).

 To sum up, revisions to these points would definitely strengthen the report's quality.

**References:**

 Reference 1

 Reference 2

 Reference 3

 Reference 4

 Reference 5

 Mudde, C. (2019). *The far right today*. John Wiley & Sons.

 Rama, J., Zanotti, L., Turnbull-Dugarte, S. J., & Santana, A. (2021). *VOX: The rise of the Spanish populist radical right*. Routledge.

 Taggart, P. (1998).

Is the case presented with sufficient detail to be useful for teaching or other practitioners?

Partly

Is the work clearly and accurately presented and does it cite the current literature?

Partly

If applicable, is the statistical analysis and its interpretation appropriate?

Not applicable

Are all the source data underlying the results available to ensure full reproducibility?

No source data required

Are the conclusions drawn adequately supported by the results?

Yes

Is the background of the case’s history and progression described in sufficient detail?

Partly

Reviewer Expertise:

My research is on political behavior and political communication with a specific focus on populism.

I confirm that I have read this submission and believe that I have an appropriate level of expertise to confirm that it is of an acceptable scientific standard, however I have significant reservations, as outlined above.

## References

[1] : Radical Left Populism from the Margins to the Mainstream: A Comparison of Syriza and Podemos.2018; 10.1007/978-3-319-63432-6_9 201-226 10.1007/978-3-319-63432-6_9

[2] : They shall not pass? The role of supply-side factors in the electoral breakthrough of VOX and Chega. *European Politics and Society* .2024;25(4) : 10.1080/23745118.2024.2303322 701-719 10.1080/23745118.2024.2303322

[3] : What's Left of the Radical Left? The European Radical Left After 1989: Decline and Mutation. *Comparative European Politics* .2005;3(1) : 10.1057/palgrave.cep.6110052 23-49 10.1057/palgrave.cep.6110052

[4] : Populist Radical Right Parties in Europe.2007; 10.1017/CBO9780511492037 10.1017/CBO9780511492037

[5] : A Touchstone of Dissent: Euroscepticism in Contemporary Western European Party Systems. *European Journal of Political Research* .1998;33(3) : 10.1023/A:1006853204101 363-388 10.1023/A:1006853204101

# Response

**Published:** 2026-07-09

**Authors:** Juan Roch

**Affiliations:** Political Science and International Relations, Autonomous University of Madrid Department of Political Science and International Relations, Madrid, Community of Madrid, Spain

Thank you for your comments. Below, we provide a point-by-point response to the issues raised.

 This country report examines the current populist actors in Spanish politics: Podemos and Vox. Please find my comments below, which, I hope, will be helpful to the authors.

 Theoretically, populism has three subdimensions: people-centrism, anti-elitism, and the Manichean outlook (Mudde, 2007). Euroscepticism can be located under anti-elitism, where European elites are depicted as self-interested outsider elites working against the pure (Spanish) people (Taggart, 1998). Both right- and left-wing populist parties carry these three characteristics. In addition, while populist radical right parties embrace populism, nativism, and authoritarianism (Mudde, 2019), populist radical left parties support a variant of socialism in which the focus on the proletariat is replaced by the vox populi (March & Mudde, 2005).

 Considering these theoretical approaches, the authors’ examination can be strengthened in several ways. First, Vox is depicted solely as a nationalist actor, which weakens its populist radical right characteristics (Rama et al., 2021). Second, the authors did not explain Vox’s rise as in-depth as Podemos’ historical trajectory. Third, the relevance of Podemos in coalition politics is explained on page 4, which can be explained earlier. Fourth, like Syriza, Podemos is a new type of party that blends socialism and populism, and is also related to discussions of 21st-century Latin American populism (Kioupkiolis & Katsambekis, 2017). Finally, once depicted as immune to exclusionary populism, Vox represents a deviation, like Chega in Portugal (Manucci, 2024).

 To sum up, revisions to these points would definitely strengthen the report's quality.

 Mudde, C. (2019). *The far right today*. John Wiley & Sons.

 Rama, J., Zanotti, L., Turnbull-Dugarte, S. J., & Santana, A. (2021). *VOX: The rise of the Spanish populist radical right*. Routledge.

 Taggart, P. (1998). **References** 1. Kioupkiolis A, Katsambekis G: Radical Left Populism from the Margins to the Mainstream: A Comparison of Syriza and Podemos. 2018; 201-226 | Publisher Full Text 2. Manucci L: They shall not pass? The role of supply-side factors in the electoral breakthrough of VOX and Chega. *European Politics and Society. *2024; **25** (4): 701-719 | Publisher Full Text 3. March L, Mudde C: What's Left of the Radical Left? The European Radical Left After 1989: Decline and Mutation. *Comparative European Politics. *2005; **3** (1): 23-49 | Publisher Full Text 4. Mudde C: Populist Radical Right Parties in Europe. 2007; | Publisher Full Text 5. Taggart P: A Touchstone of Dissent: Euroscepticism in Contemporary Western European Party Systems. *European Journal of Political Research. *1998; **33** (3): 363-388 | Publisher Full Text

 We thank the reviewer for these helpful suggestions, which have strengthened the theoretical framing of the report. We have addressed all the points raised.

 First, we have reinforced the characterisation of Vox as a populist radical-right party by explicitly incorporating its populist framing of politics as a conflict between “the people” and elites (Rama et al., 2021).

 Second, we have expanded the discussion of Vox’s emergence by adding contextual factors explaining its electoral breakthrough.

 Third, we have moved the discussion of Podemos’ role in coalition politics to an earlier section to improve the structure of the analysis.

 Fourth, we have incorporated the suggested literature on Podemos, highlighting its combination of socialism and populism and its links to Latin American populism (Kioupkiolis & Katsambekis, 2017).

 Finally, we now explicitly acknowledge that Vox’s rise represents a departure from Spain’s previous exceptionalism regarding exclusionary populist radical-right success, in line with Manucci (2024). We believe these revisions improve both the coherence and analytical depth of the report.
