# Supplementary material for: EUPopLink Country report - Spain
Source: Open Res Eur. 2026 Apr 16;6:114. [Version 1] doi: 10.12688/openreseurope.23477.1 (PMC13273243; doi:10.12688/openreseurope.23477.1)
Supplement: Peer review history

# Reviewer report: Reviewer response for version 1

**DOI:** 10.21956/openreseurope.25410.r74406

**Published:** 2026-06-16

**Authors:** Ana Belchior

**Affiliations:** 1 Universitário de Lisboa, Lisboa, Portugal

This is a pleasant-to-read and historically informative country report focusing on Euroscepticism and populism in the Spanish party system. In our view, however, there is still room for improvement. We recommend that some revisions be undertaken and also offer a few additional minor suggestions for the authors’ consideration. Our comments are listed below.

 1. A core concern relates to the balance between the treatment of populism and Euroscepticism, given that the latter clearly dominates the report. Although Euroscepticism may have a stronger and longer presence in party discourse than populism, or there may simply be more information available on this topic, if the purpose of the report is to integrate both concepts, a greater effort should be made to ensure a more balanced treatment of each. Alternatively, the authors could explicitly acknowledge that Euroscepticism has been more relevant within the Spanish party system.

 2. Another concern relates to regional parties. Since these two phenomena are not confined to Spanish national parties (e.g., Gómez-Reino et al., 2008), the authors should either explicitly acknowledge that the report focuses exclusively on national parties and therefore excludes regional parties, or they should also address Euroscepticism and populism among regional parties.

 3. We agree with the other two reviewers regarding the need for greater conceptual clarification and a clearer presentation of the dimensions underlying both concepts. For instance, the classification of parties as soft Eurosceptic requires at least a brief explanation of the soft–hard Euroscepticism dichotomy. Furthermore, if the authors choose to characterize one party in these terms, they should also position the other parties within the same framework (and maybe clarify that, in most cases, what is at stake when Euroscepticism is discussed is dissatisfaction with the performance of EU institutions rather than support for Spain's withdrawal from the EU). The same applies to populism and to the dimensions commonly associated with it in the literature, particularly in the work of Cas Mudde.

 4. The discussion of parties’ positions could also benefit from incorporating evidence from the Chapel Hill Expert Survey (CHES) on European integration and populism. For example, CHES data suggest some evolution in Podemos’ stance towards European integration over time, more clearly indicating changes in the salience attached to this issue. European integration was most salient for Podemos in 2014, declining thereafter. This pattern suggests that the politicisation of European integration was, at least in part, a strategic response to the context of the financial crisis and the political opportunities it created for a radical left populist challenger. As Blyth and Hopkin (2018) argue, the Eurozone crisis particularly favoured the emergence of radical left populist parties in Southern Europe.

 In the same vein, CHES data show VOX emerging as the Spanish party with the least favourable position towards European integration in recent years. Although the authors mention this in the conclusion, introducing this evidence earlier in the analysis would help illustrate the extent to which each of the two parties (Podemos and VOX) question European integration. Doing so would also allow for a clearer comparison between the different forms of Euroscepticism expressed by these parties. The same applies to populism.

 5. The PopuList could also be used as a consensual data source for classifying parties as populist and Eurosceptic (Rooduijn et al., 2024).

 6. A final concern relates to how the Spanish party system is situated in comparison with other European countries, especially Southern European countries, and more particularly Portugal. For some time, scholars referred to an Iberian exceptionalism regarding the absence of parliamentary representation of the populist radical right in Spain and Portugal (see, among others, Heyne and Manucci, 2021). Likewise, Portugal and Spain followed a very similar pattern in terms of party support and fluctuations in public opinion towards the EU after accession. Situating Spain within a broader comparative framework would make it possible to assess the extent to which its trajectory conforms to, or diverges from, patterns observed in other European countries.

 Other less central points:

 - The abstract could be refined to better frame the main argument regarding the presence of populism and Euroscepticism in the Spanish political system, avoiding the introduction of too many details on the historical development of both phenomena. Otherwise, the abstract reads more as a summary of the entire report, repeating what is stated later in the text. For instance, the authors could draw on the argument that Euroscepticism in Spain is mainly ephemeral and context-dependent, as suggested by several authors (e.g., Parker & Tsarouhas, 2018; Teperoglou & Belchior, 2020; or on the pandemic crisis: Belchior et al., 2026), or develop another framing that better synthesises the report’s main argument.

 - When episodes that function as historical milestones are mentioned in the report, it would be clearer if the respective dates were also included alongside them.

 - We recommend removing non-fully intuitive expressions such as “European ‘ideal’”, or clarifying what is meant by them, in order to avoid any ambiguity.

 - There is some redundancy in the development of the argument that could be reduced.

 - Finally, regarding how both mainstream parties have responded to populist challenges, the authors show that, despite ideological nuances, both the PSOE and PP have adopted an accommodative approach. With regard to their stance on EU integration, the authors clearly demonstrate that both parties not only maintained their positions but even continued to advocate for the deepening of European integration. In highlighting this aspect, the authors appear to suggest that this outcome was somewhat expected, and that it did not materialise. As they note, “however, what is particularly striking is that the rise of these populist and Eurosceptic forces has not led the mainstream parties to alter their pro-EU stance”. It may also be worth considering the possibility that, by maintaining their commitment to EU integration, the PSOE and PP seek to preserve their image as responsible governing parties, without jeopardising their position among electorates that continue to support the European project.

**References:**

 Reference 1

 Reference 2

 Reference 3

 Reference 4

 Gómez-Reino M, Llamazares I and Ramiro L (2008) Euroscepticism and political parties in Spain. In: Szczerbiak A and Taggart P (eds) *Opposing Europe? The Comparative Party Politics of Euroscepticism volume 1: Case Studies and Country Surveys.* Oxford: Oxford University Press, pp.134–151.

 Parker, O., & Tsarouhas, D. (Eds.). (2018). *Crisis in the Eurozone periphery, The political economies of Greece, Spain, Ireland and Portugal*. Springer.

 Rooduijn, Matthijs, Andrea L.P. Pirro, Daphne Halikiopoulou, Caterina Froio, Stijn van Kessel, Sarah L. de Lange, Cas Mudde, and Paul Taggart (2024). The PopuList: A Database of Populist, Far-Left, and Far-Right Parties Using Expert-Informed Qualitative Comparative

 Classification (EiQCC). *British Journal of Political Science*, 1–10 **.**

Is the case presented with sufficient detail to be useful for teaching or other practitioners?

Partly

Is the work clearly and accurately presented and does it cite the current literature?

Yes

If applicable, is the statistical analysis and its interpretation appropriate?

Not applicable

Are all the source data underlying the results available to ensure full reproducibility?

No source data required

Are the conclusions drawn adequately supported by the results?

Partly

Is the background of the case’s history and progression described in sufficient detail?

Yes

Reviewer Expertise:

Political Science; including Euroscepticism and Populism

I confirm that I have read this submission and believe that I have an appropriate level of expertise to confirm that it is of an acceptable scientific standard, however I have significant reservations, as outlined above.

## References

[1] : Euroscepticism in party discourse during the COVID-19 pandemic: Evidence from Portugal and Spain. *European Union Politics* .2026;27(2) : 10.1177/14651165261424975 373-394 10.1177/14651165261424975

[2] : The Global Economics of European Populism: Growth Regimes and Party System Change in Europe (TheGovernment and Opposition /Leonard Schapiro Lecture 2017). *Government and Opposition* .2019;54(2) : 10.1017/gov.2018.43 193-225 10.1017/gov.2018.43

[3] : A new Iberian exceptionalism? Comparing the populist radical right electorate in Portugal and Spain. *Political Research Exchange* .2021;3(1) : 10.1080/2474736X.2021.1989985 10.1080/2474736X.2021.1989985

[4] : Ιs ‘Old Southern Europe’ Still Eurosceptic? Determinants of Mass Attitudes before, during and after the Eurozone Crisis. *South European Society and Politics* .2024;29(4) : 10.1080/13608746.2020.1805878 405-430 10.1080/13608746.2020.1805878
